# Examining the influence of pain neuroscience education followed by a Pilates exercises program in individuals with knee osteoarthritis: a pilot randomized controlled trial

**DOI:** 10.1186/s13075-023-03079-7

**Published:** 2023-06-06

**Authors:** Pouya Rabiei, Bahram Sheikhi, Amir Letafatkar

**Affiliations:** 1 Faculty of Medicine, Université Laval, Quebec City, Canada; 2 Centre Interdisciplinaire de Recherche en Réadaptation Et Intégration Sociale (Cirris), 525 Boulevard Hamel, Quebec, QC G1M 2S8 Canada; 3 Sports Injury and Corrective Exercises, Faculty of Physical Education and Sports Sciences, Kharazmi University, Tehran, Iran

**Keywords:** Kinesiophobia, Knee osteoarthritis, Pain catastrophizing, Pain neuroscience education, Pilates

## Abstract

**Background:**

Knee osteoarthritis (OA) is a multifactorial form of rheumatic condition contributing to physical and psychological factors. Treatments have been provided solely and often compared with each other. An alternative view is that combined treatments addressing physical and psychological factors may result in more benefits. This study aimed to investigate the effect of pain neuroscience education (PNE) followed by Pilates exercises (PEs) in participants with knee OA, compared to PE alone.

**Methods:**

In this two-arm assessor-blind pilot randomized controlled trial, fifty-four community-dwelling adults with knee OA were randomly assigned to the PNE followed by PEs and PEs groups (27 in each group). The study was conducted between early July 2021 and early March 2022 at the university’s health center. Primary outcomes were Western Ontario and McMaster Universities Osteoarthritis Index (WOMAC) subscales of pain and physical limitation and secondary outcomes were Pain Catastrophizing Scale, Tampa Scale for Kinesiophobia, Pain Self-Efficacy Questionnaire, and Timed "Up & Go" test covering function. The primary and secondary outcomes were measured at baseline and eight weeks post-treatment. A general linear mixed model was used for between-group comparison with a statistical significance level of 0.05.

**Results:**

Significant within-group differences were observed in all outcomes in both groups at post-treatment. There were no statistically between-group differences in pain (adjusted mean difference: -0.8; 95% CI -2.2 to 0.7; *p* = 0.288), physical limitation (adjusted mean difference: -0.4; 95% CI -4 to 3.1; *p* = 0.812) and function (adjusted mean difference: -0.8; 95% CI -1.8 to 0.1; *p* = 0.069) at eight weeks. For pain catastrophizing (adjusted mean difference: -3.9; 95% CI -7.2 to -0.6; *p* = 0.021), kinesiophobia (adjusted mean difference: -4.2; 95% CI -8.1 to -0.4; *p* = 0.032), and self-efficacy (adjusted mean difference: 6.1; 95% CI 0.7 to 11.5; *p* = 0.028) statistically between-group improvements were observed favoring PNE followed by PEs group after the treatment.

**Conclusions:**

Combining PNE with PEs could have superior effects on psychological characteristics but not on pain, physical limitation, and function, compared to PEs alone. This pilot study emphasizes the need to investigate the combined effects of different interventions.

**Trial registration:**

IRCT20210701051754N1.

## Background

Osteoarthritis (OA) is a multifactorial widespread chronic rheumatic condition leading to difficulty performing daily activities [1] and psychosocial factors which contribute to prolonged activity [2], pain chronification [3, 4], and treatment effectiveness [5]. In addition to pain and physical disability, pain catastrophizing, self-efficacy, and kinesiophobia are the expected consequences of knee OA [3, 6]. In understanding clinical features related to knee OA, such as physical limitation, psychological factors might offer something additional beyond what might be explained by traditional factors, underscoring the importance of multidisciplinary treatment approaches in knee OA management [7].

Different therapeutic exercise programs (e.g., aerobic, strengthening, or a combination of different types of exercise) have been introduced to improve knee OA [8, 9]. To date, studies have investigated the influence of Pilates exercises (PEs) on the improvement of rheumatological conditions [10, 11]. PEs, unlike traditional programs, affects strength, cardiovascular and respiratory function, and additional benefits, including enhanced coordination, proprioceptive acuity, and state of well-being [12, 13]. Oksuz et al. (2017) reported that a clinical PEs, based on cognitive behavioral therapy, can positively affect kinesiophobia, pain, and functional status in individuals with osteoporosis [14]. Similarly, a previous investigation showed the superiority of PEs over knee-strengthening exercises in improving pain and disability in subjects with knee OA [15]. However, there is not yet adequate evidence showing that PEs includes a clear and specific focus on psycho-cognitive components of pain [16], which necessitates adding a psychological-based approach.

Pain neuroscience education (PNE) has been introduced as an effective intervention for improving patients' knowledge of pain, reducing pain and central sensitization and improving psychological factors [17]. This educates the subject on the mechanism behind chronic pain and reframes unhelpful and negative beliefs about pain [18, 19]. Watson et al. (2019) concluded that PNE can reduce pain, disability, pain catastrophizing, and kinesiophobia in the short to medium term in those with chronic musculoskeletal pain [20]. On the other hand, Lluch et al. (2018) reported that although PNE combined with knee joint mobilization did not produce any additional benefits for knee pain and disability in knee OA patients, it effectively reduced pain catastrophizing and kinesiophobia [21]. Allowing the participants to tell their own story,provided in PNE sessions, can be a key component of improving the individual experience of pain education and pain reconceptualization, which seems to be an important process to facilitate participants’ ability to cope with their condition [20].

Prescribing exercise therapy and education were the most recommended interventions for improving musculoskeletal pain [13, 22–24]. Moreover, using pain education as an isolated intervention has resulted in 45% less health care expenditure, and its effect was maintained after three years [25, 26]. However, it seems that pain management approaches are not yet popular to use by physical therapists in clinical settings [27]. Besides, there is a lack of knowledge in the prescription of pain management, making pain a bigger barrier in areas where knowledge is lacking [27]. Thus, emphasizing the importance of education and effective therapeutic exercise(s) must be highlighted.

Therefore, we aimed to conduct a randomized controlled trial (RCT) to provide pilot data investigating the effect of PNE followed by PEs in those who suffered from knee OA, compared to PEs alone. We hypothesized that participants receiving PNE followed by PEs would have superiority over those in PEs group to reduce pain and physical limitation, improve psychological factors, and improve function through eight weeks post-treatment.

## Methods

### Study’s design and population

This was a two-arm, assessor-blind pilot RCT performed between early July 2021 and early March 2022. The study was approved by the ethical committees of the Sport Sciences Research Institute (IR.SSRC.REC.1400.033) and prospectively registered at www.irct.ir (IRCT20210701051754N1). The study was reported following the Consolidated Standards of Reporting Trials (CONSORT) guideline [28], and all experimental conditions conformed to the Declaration of Helsinki [29]. All participants provided written informed consent before enrolment.

Participants were recruited through flyers in physical therapy clinics, social media, and adverts. Based on the inclusion criteria, eligible participants were enrolled to participate in the study. Of 92 enrolled participants, 54 met the criteria to enter the study. Participants were eligible to participate if they: were Persian-native speakers males and females complained of knee pain diagnosed as chronic tibiofemoral joint OA (> 3 months). The American College of Rheumatology classification system, with 91% sensitive and 86% specific, was used for a diagnosis of tibiofemoral joint OA if a person has knee pain and osteophytes confirmed by radiography with the following conditions: experiencing stiffness for less than 30 min in the morning, having crepitus, and being older than 50 years of age [30]. Additionally, OA grade 2 or 3 on the Kellgren/Lawrence classification based on plain radiographs [31], as evaluated by an orthopedic physician with more than 10 years of clinical experience, has been considered. Exclusion criteria were: other forms of arthritis than OA (e.g., crystal arthropathy, septic arthritis, spondyloarthropathy) identified by radiography, presence of comorbidity resulting in severe activity limitations, total knee arthroplasty (TKA) or TKA shortly, severe knee pain > 80 in a 0–100 Visual Analog Scale, knee ligament or meniscus injury in the previous year, any mental health conditions, and therapeutic modalities six months before participation, [21, 32].

### Randomisation and blinding

Following the baseline examination, by using the method on the website http://randomizer.org/ (Social Psychology Network, Connecticut, USA), participants were randomly assigned to the PNE, followed by the PEs group and PEs group. In a simple randomisation, the concealed allocation was performed using a computer-generated block randomized table of numbers created before the start of data collection by a researcher who was not involved in the recruitment or treatment of participants. Block randomization was performed to ensure balance in the number of participants between the groups (block size of 12 participants). Then, the random numerical sequence was placed in sealed opaque envelopes. Next, another researcher opened an envelope and processed with treatment according to the group assignment.

### Experimental procedures

The participants were assessed within a week before the intervention (baseline) and after an eight-week intervention (post-treatment) by a blind assessor with over seven years of experience. The primary outcomes were pain and physical limitation, and the secondary outcomes were pain catastrophizing, kinesiophobia, self-efficacy, and function. Participants were asked to complete the indexes for demographic information, pain, physical limitation, pain catastrophizing, kinesiophobia, and self-efficacy online. At the same time, the function assessment was performed in person at the biomechanics laboratory.

### Outcome measures

*Pain and physical limitation*. Persian version of the Western Ontario and McMaster Universities Osteoarthritis (WOMAC) index is a valid and reliable (ICC = 0.63 to 0.94) tool for assessing pain and physical limitation in OA participants [33, 34]. It has 5 items for measuring pain and 17 items for physical limitation. The test questions are scored on a scale of 0–4, which correspond to none (0), mild (1), moderate (2), severe (3), and extreme (4). The scores for each subscale are summed up, with a possible score range of 0–20 for pain and 0–68 for physical limitation. For pain, participants are asked to indicate their pain level during walking, using stairs, in bed, sitting or lying, and standing upright. For physical limitation, participants need to score their level of limitation while using stairs, rising from sitting, standing, bending, walking, getting in/out of a car, shopping, putting on / taking off socks, rising from bed, lying in bed, getting in/out of the bath, sitting, getting on / off toilet, heavy domestic duties, light domestic duties [34].

*Pain Catastrophizing*. Pain Catastrophizing Scale (PCS) consists of 13 items scored on a five-point Likert scale and measures aspects of catastrophic cognitions about pain-rumination that individuals may have when experiencing pain. Higher scores indicate more severe catastrophic thoughts about pain [35]. The reliability of the Persian version of PCS has been previously confirmed (ICC = 0.93) [35].

*Kinesiophobia*. Tampa Scale for Kinesiophobia (TSK) contains 17 items related to fear of movement and re-injury. The score ranges from 17 to 68 (scores ≤ 37 presenting low fear and scores > 37 presenting high fear of movement) [36]. Persian version of TSK has been previously reported as valid and reliable (ICC = 0.77 to 0.78) [36].

*Self-efficacy*. The Persian version of the Pain Self-Efficacy Questionnaire (PSEQ) was used to assess self-efficacy as a valid and reliable questionnaire (ICC = 0.92) [37]. {Asghari, 2009 #17;Asghari, 2009 #14} The PSEQ is a 10-item questionnaire ranging from 0 to 60 to assess participants’ confidence in their ability to perform various activities despite pain. For example: “I can do most of the household chores (e.g., tidying up, washing dishes), despite the pain”, and “I can gradually increase my activity level, despite the pain. Lower scores for the PSEQ indicate lower levels of confidence [37].

*Function*. The Timed "Up & Go" (TUG) test is a valid and reliable test extensively used for assessing function in OA participants [38] with ICC = 0.95–0.97 [39]. The chair height used for all tests was 41 cm, and the time required to complete the TUG was recorded in seconds. Participants needed to rise from their chair without using the hand rests; walk as quickly as they could over 3 m, marked by a line of tape; turn around once they crossed the tape; return to the chair; and sit down. Participants did not have to use gait aids during the TUG or other tests [40].

### Interventions

*Pain neuroscience education*. Before participating in PEs, participants in the PNE, followed by the PEs group, took part in three PNE sessions held by a licensed native-speaker physical therapist (A) with more than five years of experience and knowledge about pain science and exercise therapy. Each PNE session lasted approximately 30 to 60 min for each participant, and the topic(s) for each session was based on the practice guideline developed by Nijs et al. (2011) [41]. The main aims of PNE were to reframe the participant's unhelpful and negative beliefs about pain and decrease the threatening feeling of pain by providing the participant with information about the nature of pain. These beliefs might have been imposed by potentially unhelpful diagnostic, prognostic, or therapeutic conclusions in the participant's mind. In PNE, providing the subjects with information about the nature of pain was targeted to reduce fear avoidance and avoidance behavior and to increase self-efficacy [18]. PNE includes the important points in nontechnical terms: neurophysiology of pain, peripheral sensitization, and central sensitization delivered using verbal instructions, questions and answers, pictures, and free-hand drawings [42, 43]. As most of the participants had no educational background in pain mechanisms, and it was the first time they received such information, a good-quality voice record of what was discussed at the education sessions with related pictures was provided for participants. Participants were recommended to listen to the record and check the diagrams whenever needed. This process could help the participants better understand what they were taught.

*Pilates exercise*. Participants in each treatment group received exercises in group sessions. Participants in both groups received PEs for 24 sessions (eight weeks, three sessions each week) designed based on our previous study [15]. Participants who received PNE continue their PEs on odd days by the same physical therapist (A) who has applied PNE. Using the same supervisor could help participants better communicate with their supervisor, and re-receive the important elements discussed in PNE sessions (e.g., decreasing threat level, assuring the safety of the exercises, and increasing confidence in a successful accomplishment of the exercise). Those in the PEs group received their training on even days via another physical therapist (B) who was a native speaker, knowledgeable about PEs and had three years of experience. Each session took 60 min (10 min for warm-up, 40 min for PEs [with a gradual increase from 20 min], and 10 min for cool-down). The number of repetitions started from five and gradually increased according to the participant's ability (Table 1).

**Table 1:** Pilates exercises protocol

Week	The exercises	Explanation	Frequency
1^st^ week	Hundred 1/2/3	Lying on the back with your knees bent and up in the air, then position your knees and hips at 90° angles	3 × 20 s
2^nd^ week	The 1st-week exercises + One leg stretch 1, Double leg stretch, 1/2, Clam	One leg stretch: Lying on the floor with legs extended straight out in front which let the belly drop toward the floor	3 × 20 s
		Double leg stretch: Lying on the floor with legs extended straight out in front. Press your legs together and point your toes in the Pilates stance and let the belly drop toward the floor	
		Clam: Lying on the side. Resting the head on the arm or hand, then bend hips to approximately 45 and knees at 90°. Make sure that one hip is lying above the other. Upper leg upwards while keeping the feet in contact with one another	3 × 5 repetitions with an increase based on the participant's ability
3^rd^ week	The 2nd-week exercises + One leg stretch 2, Shoulder Bridge 1	Lying on the back in neutral spine, with knees bent and feet on the floor. Come to a bridge position on shoulders with knees, hips, and shoulders in one line. Your abs and hamstrings should be well engaged	3 × 5 repetitions with an increase based on the participant's ability
4^th^ week	The 3rd-week exercises + Shoulder bridge 2, Hip Twist	Sitting on a mat and extending your legs in front. The body should be in the same position as a V-up. Rotate the hips to the right side of the body and circle the legs to the right and down	3 × 5 repetitions with an increase based on the participant's ability
5^th^ week	The 4th-week exercises + Scissors 1, One leg kick	In the prone position, close the back of the legs to the same leg hamstring and firm the buttock while increasing your abdominal strength. If you have tight quads, only bend your knee as far as is comfortable, or feel free to pulse only once	3 × 20 s
6^th^ week	The 5th-week exercises + Scissors 2, Sidekick 1	Lying on the side and lining up ears, shoulders, hips, knees, and ankles. Then, abduct the upper leg	3 × 5 repetitions with an increase based on the participant's ability
7^th^ week	The 6th-week exercises + Sidekick 2, One leg circle 1/2	Lying on the back with legs extended. Bring your thigh as perpendicular to the floor as possible Circle the leg down toward the mat, then out to the right, and finally back to the center	3 × 5 repetitions with an increase based on the participant's ability
8^th^ week	The 7th-week exercises	-	3 × 5 repetitions with an increase based on the participant's ability

PEs were deisgned based on six main principles. (1) Centering: physically bringing the focus to the center of the body to provide good protection for the spine and trunk and pass on power to each movement, (2) Control: doing every exercise with complete and conscious muscular control, (3) Precision: considering doing the exercise correctly and sustaining awareness throughout each movement, (4) Concentrating: bringing full attention to the exercise and doing them with full commitment, (5) Breath: being prepared by inhaling for the performing motion and exhale operation to move, activating trunk muscle and intensifying movement, and (6) Flow: doing exercises in a flowing manner, connecting the energy of an exercise to all body parts and flowing through the body in an even way [15, 44].

### Sample size calculation

Sample size calculations were performed using G*Power (version 3.1.9.2, Dusseldorf, Germany). The calculations were based on detecting differences of 20 units in the pain intensity, considered as the minimum clinically important difference (MCID), assuming a standard deviation of 17 (based on previous studies [45, 46]). A medium effect size (f = 0.25), an alpha level of 0.05, and a power of 0.80 were considered [47, 48]. The calculation revealed that 22 participants were required in each group. To account for possible missing data and a 20% loss from participants missing follow-ups, 27 participants were included in each group.

### Statistical analysis

Data were analysed using SPSS-26. Kolmogorov–Smirnov was used for the normal distribution of the outcomes. The homogeneity of the variations was observed using the Levene test. A general linear mixed model was used to compare outcome measures between groups over time (baseline and post-treatment) and group effects (PNE followed by PEs and PEs groups). Effect sizes and 95% confidence intervals (CIs) were calculated to measure clinical meaningfulness. Effect sizes were expressed in partial eta squared (*η* $$\begin{array}{c}2\\ p\end{array}$$), with values of 0.01, 0.06, and 0.14 representing small, medium, and large effects, respectively [49]. The group replaced for intention-to-treat (ITT) analyses, missing baseline data means. The psychological characteristics (pain catastrophizing, kinesiophobia, and self-efficacy) were independent variables. Statistical significance was set at an α level of < 0.05.

## Results

### Study’s population

**Fig. 1:**
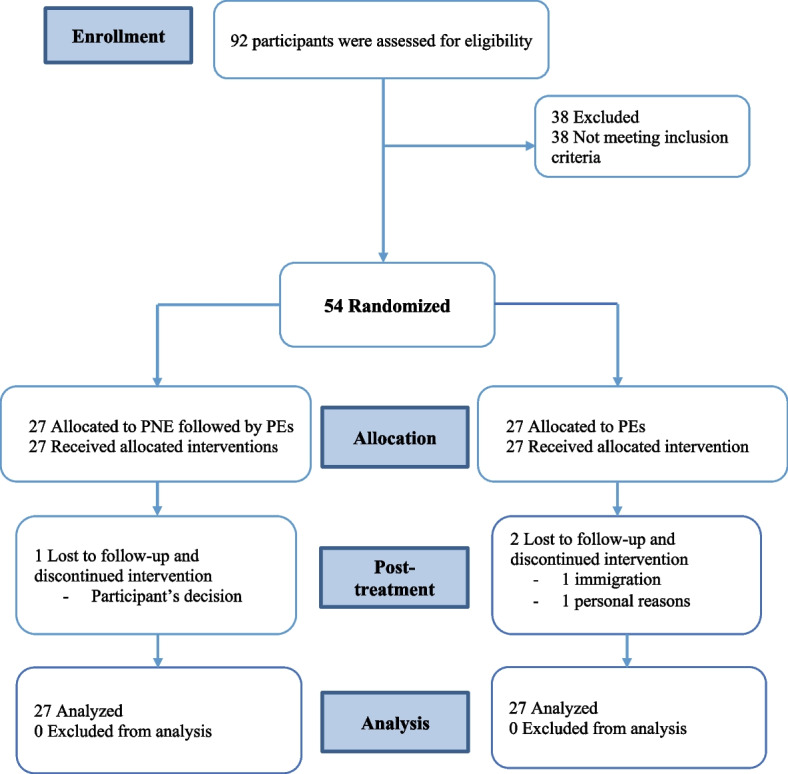
CONSORT Flow Diagram

In total 54 participants with knee OA were recruited. The baseline characteristics of the two groups were similar for demographic, and primary and secondary outcomes measures (Tables 2 and 3). Of these, three did not complete the study (Fig. 1). Compliance was 96.3% for the participants of PNE followed by PEs group and 92.6% for PEs group. No serious adverse events were reported in any of the intervention groups. Protocol deviations or adjustments did not occur for both group.

**Table 2:** Baseline demographic data by intervention group

Characteristic	Total sample (*n* = 54)	PNE followed by PEs (*n* = 27)	PEs (*n* = 27)
Age, y	60.5 ± 5.6	59.8 ± 5.1	61.2 ± 6.1
Body height, cm	166.2 ± 6.5	167.3 ± 5.3	164.7 ± 7.3
Body mass, kg	81.2 ± 10.6	82.1 ± 10.1	80.1 ± 11.2
Body mass index, kg/m^2^	29.5 ± 4.4	29.3 ± 3.4	29.7 ± 5.3
Sex, n (%)			
Female	22 (40.7)	9 (40.9)	13 (59.1)
Male	32 (59.3)	18 (56.3)	14 (43.8)
VAS pain rating (0–100)	54.1 ± 13.2	56.3 ± 13.3	51.7 ± 12.8
Pain duration, y	7.8 ± 4.5	7.6 ± 4.7	6.7 ± 4.0
Unilateral symptoms, n (%)	13 (24.1)	8 (29.6)	5 (18.5)
Smoking status, n (%)			
Never smoked	36 (66.7)	17 (63.0)	19 (70.4)
Current	9 (16.7)	5 (18.5)	4 (14.8)
Past	9 (16.7)	5 (18.5)	4 (14.8)
Education level, n (%)			
High school or less	27 (50)	12 (44.4)	15 (55.6)
Bachelor’s degree	18 (33.3)	10 (37)	8 (29.6)
Master’s degree or higher	9 (16.7)	5 (18.5)	4 (14.8)
Marital status, n (%)			
Married	37 (68.5)	17 (63.0)	20 (74.1)
Single	3 (5.6)	2 (7.4)	1 (3.7)
Separated/divorced/widowed	14 (25.9)	8 (29.6)	6 (22.2)

*Abbreviations*: Continuous variables were expressed as mean and standard deviation (SD) and categorical variables as number (n) and percentage (%), *VAS* Visual Analog Scale; *PEs* Pilates exercises, *PNE followed by PE* Pain neuroscience education followed by Pilates exercises

### Pain and physical limitation

There was no statistically significant or clinically important difference between groups in the primary outcome, pain (adjusted mean difference of -0.8; 95% CI -2.2 to 0.7; *p* = 0.288) and physical limitation (adjusted mean difference of -0.4; 95% CI -4 to 3.1; *p* = 0.812). In PNE followed by PEs group, pain, and physical limitation were respectively reduced by 31.1% and 21.7%, compared to PEs group 24.3% and 18.9% (Table 3).

### Psychological characteristics

For the secondary outcomes, the pain catastrophizing (adjusted mean difference of -3.9; 95% CI -7.2 to -0.6; *p* = 0.021), kinesiophobia (adjusted mean difference of -4.2; 95% CI -8.1 to -0.4; *p* = 0.032), and self-efficacy (adjusted mean difference of 6.1; 95% CI 0.7 to 11.5; *p* = 0.028) revealed a statistically significant between-group difference, favoring the PNE followed by PEs group at 8-weeks after treatment. This amounted to a 21.5% reduction in kinesiophobia for the PNE followed by PEs group and a 10.3% decrease for PEs group. Pain catastrophizing in PNE followed by PEs group reduced by 37.9% and declined by 19.3% in PEs group. The greatest PNE followed by PEs group improvements were for self-efficacy, by 40.5%, while PEs group scores increased by 21.5% (Table 3).

### Function

Participants with a prior preference for the PNE followed by PEs might potentially benefit from the PNE followed by PEs after treatment compared to those with a preference for PEs with a difference of -0.8 function points (adjusted mean difference -0.8; 95% CI -1.8 to 0.1; *p* = 0.069) that did not reach statistical significance. In PNE, followed by PEs group, function improved by 24%, while it was 17.2% in PEs group (Table 3).

**Table 3:** Within- and between-group differences in primary and secondary outcome measures based on the general linear mix model analysis

Variables	Group	Baseline Mean (SD)	Eight weeks Mean (SD)	Change relative to baseline (%)	Group Difference, Mean (95% CI)	ES (*η*_*p*_^2^)^†^	*P*-value
Pain (0–20)	PNE followed by PEs	10.6 (2.8)	7.3 (2.3)	-31.1	-0.8 (-2.2 to 0.7)	0.04	0.288
	PEs	10.7 (3.2)	8.1 (2.9)	-24.3			
Physical limitation (0–68)	PNE followed by PEs	29 (8.4)	22.7 (7.2)	-21.7	-0.4 (-4 to 3.1)	0.02	0.812
	PEs	28.5 (7.5)	23.1 (5.9)	-18.9			
Pain catastrophizing (0–52)	PNE followed by PEs	26.1 (7.2)	16.2 (5.6)	-37.9	-3.9 (-7.2 to -0.6)	0.51^¥^	0.021
	PEs	24.9 (8)	20.1 (6.5)	-19.3			
Kinesiophobia (17–68)	PNE followed by PEs	43.7 (7.8)	34.3 (7.3)	-21.5	-4.2 (-8.1 to -0.4)	0.39^¥^	0.032
	PEs	42.9 (7.5)	38.5 (6.8)	-10.3			
Self-efficacy (0–60)	PNE followed by PEs	34.1 (7.5)	47.9 (7.2)	40.5	6.1 (0.7 to 11.5)	0.13	0.028
	PEs	34.4 (11.8)	41.8 (12.0)	21.5			
Function (s)	PNE followed by PEs	12.1 (2)	9.2 (1.6)	-24	-0.8 (-1.8 to 0.1)	0.05	0.069
	PEs	12.2 (2.1)	10.1 (1.8)	-17.2			

*Abbreviations*: †, Effect size (partial eta squared); ¥, Large effect size (0.14), *CI* Confidence Interval, *PEs* Pilates exercises, *PNE followed by PE* Pain neuroscience education followed by Pilates exercises

## Discussion

Based on the findings, the first study’s hypothesis regarding the effect of PNE followed by PEs on pain and physical limitation was not accepted. Although clinical effectiveness was larger in the group of PNE followed by PEs, no statistically significant differences were observed between the two groups. For the second hypothesis, PNE followed by the PEs group showed only statistically and clinically significant differences compared to the PEs group in psychological outcomes. Finally, no differences were found in the outcome of the function.

The effectiveness of prescribing PNE with therapeutic exercise is controversial. Two systematic review studies reported low clinical benefits of PNE in combination with other short- or medium-term therapeutic exercises for reducing pain and disability [20, 50]. Inconsistently, a recent review study found significant differences in pain, disability, kinesiophobia, and pain catastrophizing that favored the combination of PNE and exercise [51].

To support our results, Lluch et al. (2018) concluded that a preoperative program including PNE (similar to the pain education format of our study) and knee joint mobilization has a superior effect on pain catastrophizing and kinesiophobia over biomedical education and knee joint mobilization in knee OA participants, while, no differences were found for pain and disability [21]. Contrary to our findings, Ryan et al. (2010) showed the short-term superiority of pain biology education over combined pain biology education and group exercise classes for improving of pain and pain self-efficacy in chronic low back pain participants [52]. The main differences between Ryan et al. study and ours were related to the type of pain location (low back pain versus knee OA) and exercise interventions. Ryan et al. provided group-based exercise focusing on strengthening, stretching, and cardiovascular fitness, 1 session each week for 6 weeks (overall 6 sessions of exercise), while we provided 24 sessions of PEs during 8 weeks. Thus, types of exercise and different dosages in a supervised manner may be the reasons behind the effectiveness of PEs with and without supplementary pain education sessions.

Alongside this, one explanation for the lack of statistical effectiveness of PNE in reducing pain can be related to the number of educational sessions. Whereas in the current study, PNE was provided for three PNE sessions, the previous investigation showed that a higher dosage (six 45-min sessions) produced a larger improvement in pain [53]. A higher dosage of educational sessions may provide the possibility of introducing and discussing new concepts and giving more time to teach and assimilate the concepts taught [53]. In addition, the measurement of physical limitation and function was based on daily physical activities. It is speculated that PEs as a movement-based intervention could directly influence these outcomes instead of PNE, which is a psychological-based approach.

Regarding psychological outcomes, one-way pain education could be effective and increase the benefits of PEs associated with its potential to reduce the threat value of pain leading to fear of movements. This is observed that the effectiveness of pain education can achieve by targeting a clear and valid explanation of pain and symptoms in chronic pain participants [43] and improving participants’ understanding of pain by overcoming traditional beliefs (that pain results from and is related to tissue damage) toward a modern view that pain can be the result of central sensitization. In contrast, the real damage has been healed [54]. This improvement in participants' understanding and knowledge of pain can facilitate recovery by reconceptualizing the participant’s belief from pain control toward being active, which might have been avoided due to the fear of pain.

Unlike the current results, our previous study showed that individualized PNE combined with motor control training has no additional benefits for pain and disability and psychological factors (e.g., fear-avoidance beliefs and self-efficacy) compared to a group-based intervention in participants with chronic low back pain [55]. As the pain education program in both studies followed the same targets, the different results can be related to the pain location/types and the added exercises. Here, we used PEs to improve flexibility, general body perception and awareness, and activate lower limb muscles. As lower limb muscles’ weakness (gluteal, quadriceps, and hamstring) and reduced proprioceptive acuity are potentially modifiable putative risk factors for knee OA [56, 57], we can speculate that PEs could be a more appropriate movement-based intervention to be added with a psychological one, both leading to better results.

One unique procedure we used in the group of PNE followed by PEs was providing the same supervisor (physical therapist A) for both educational pain classes and PEs sessions. We believe this procedure may benefit participants via 1. receiving the same information from the same supervisor, 2. reminding pain education topics while doing PEs, making the educational classes into practice, and 3. better communication of participants with the allocated supervisor during the interventions. In a study by Ryan et al. (2010), the superiority of pain biology education over combined pain biology education and group exercise classes was mentioned because of “mixed information” provided by the supervisors for participants in the combined group [52]. The authors also stated that using different information may have led to confusion, and even frustration, on the participant's part, which could have negatively impacted participant improvement [52]. To support such an idea, Little et al. (2001) concluded that providing different amounts and formats of information can lead to poorer outcomes than using one set alone [58]. Although we tried to overcome mixed information, further investigations are still required to test it.

### Strengths and limitations

A unique quality of this study is that the study has been undertaken outside Europe/USA/Australia, where most of the pain science education studies have been carried out. This work in a non-western culture provides a unique insight into the potential effectiveness of PNE on a more global level.

However, this is not without limitations. The first is related to blinding. In this study, participants were not blinded to the group allocation, and given measures of pain, physical limitation, and psychological outcomes were answered via participants (participants being the assessors). So, we can only claim that the assessor was blinded to the outcome of the function. The second is related to a small proportion of participants. Although the number of participants in each group was based on G*Power analysis, larger samples would have led to more precise estimates and reduced risk of a type II error. The third is the lack of evaluating attentional control of the participant's time spent with the physical therapist. For example, in the PNE followed by the PEs group, participants interacted with their pain education physical therapist (A), even in the PEs sessions. These interaction has been previously investigated and shown important in treatment response [59].

## Conclusions

Compared to PEs alone, combining PNE with PEs could have only superior effects on psychological characteristics but not on pain, physical limitation, and function. Clinical effectiveness was more significant in the combined group for all study outcomes. This pilot RCT emphasizes the need to investigate the combined effects of different interventions, including education and exercise therapy, in the format of full-scale studies.

## Data Availability

The datasets used and/or analyzed during the current study are available from the corresponding author upon reasonable request.

## Abbreviations

OA: Osteoarthritis
PEs: Pilates exercises
PNE: Pain neuroscience education
RCT: Randomized controlled trial
CONSORT: CONsolidated Standards of Reporting Trials
WOMAC: Western Ontario and McMaster Universities Osteoarthritis
PCS: Pain Catastrophizing Scale
TSK: Tampa Scale for Kinesiophobia
PSEQ: Pain Self-Efficacy Questionnaire
TUG: Timed "Up & Go"
MCID: Minimum clinically important difference
ITT: Intention-to-treat
